# Transcriptome Profiling Based on Larvae at Different Time Points After Hatching Provides a Core Set of Gene Resource for Understanding the Metabolic Mechanisms of the Brood-Care Behavior in *Octopus ocellatus*

**DOI:** 10.3389/fphys.2021.762681

**Published:** 2022-01-07

**Authors:** Xiaokai Bao, Xiumei Liu, Benshu Yu, Yan Li, Mingxian Cui, Weijun Wang, Yanwei Feng, Xiaohui Xu, Guohua Sun, Bin Li, Zan Li, Jianmin Yang

**Affiliations:** ^1^School of Agriculture, Ludong University, Yantai, China; ^2^College of Life Sciences, Yantai University, Yantai, China; ^3^Shandong Fisheries Development and Resources Conservation Center, Yantai, China; ^4^Yantai Haiyu Marine Science and Technology Co. Ltd., Yantai, China

**Keywords:** *Octopus ocellatus*, transcriptome, protein–protein interaction networks, metabolism, brood-care behavior

## Abstract

The metabolic processes of organisms are very complex. Each process is crucial and affects the growth, development, and reproduction of organisms. Metabolism-related mechanisms in *Octopus ocellatus* behaviors have not been widely studied. Brood-care is a common behavior in most organisms, which can improve the survival rate and constitution of larvae. *Octopus ocellatus* carried out this behavior, but it was rarely noticed by researchers before. In our study, 3,486 differentially expressed genes (DEGs) were identified based on transcriptome analysis of *O. ocellatus*. We identify metabolism-related DEGs using GO and KEGG enrichment analyses. Then, we construct protein–protein interaction networks to search the functional relationships between metabolism-related DEGs. Finally, we identified 10 hub genes related to multiple gene functions or involved in multiple signal pathways and verified them using quantitative real-time polymerase chain reaction (qRT-PCR). Protein–protein interaction networks were first used to study the effects of brood-care behavior on metabolism in the process of growing of *O. ocellatus* larvae, and the results provide us valuable genetic resources for understanding the metabolic processes of invertebrate larvae. The data lay a foundation for further study the brood-care behavior and metabolic mechanisms of invertebrates.

## Introduction

Cephalopods are marine mollusks that are widely distributed throughout the world’s oceans. The artificial breeding of cephalopods has gradually appeared all over the world in the last few years because of the difficult catching (Arkhipkin, 1985; Budelmann, 1994; Schnell et al., 2020). Previous studies have shown that the reproductive cycle of cephalopods is generally around 2 months (Zheng et al., 2009; Wang et al., 2010). They lay eggs about a month after mating, and the eggs hatch after 25–40 days. It’s worth noting that water temperature significantly affects the incubation time, suitable water temperature will greatly reduce the incubation time (Wang et al., 2010; Zheng et al., 2011). Normally, the incubation rates of cephalopod larvae are 70–80%, and the survival rate is more than 90% (Zheng et al., 2009, 2011; Wang et al., 2010). *Octopus ocellatus* has become a sought-after breed for its high nutritional value, short growth cycle, rapid growth, strong adaptability to the breeding environment, and ease of reproduction (Boletzky, 1975; Navarro and Villanueva, 2003; Rosas et al., 2013; Wei et al., 2015). In China, the research on *O. ocellatus* larvae has mainly focused on survival, feeding, and palatability factors, with relatively less research on the breeding methods of *O. ocellatus* during the hatching period (Boletzky, 1989; Li et al., 2019).

There are many factors that affect the growth and development of *O. ocellatus* larvae, including water quality, dissolved oxygen, light, temperature, and brood-care (Boletzky, 1989; Quiroga and Garcia, 2003; Huffard, 2013; Vidal et al., 2014; Seibel, 2016; Chatterjee et al., 2018). The brood-care behavior of female *O. ocellatus* can reduce the occurrence of hatching diseases and improve the hatching rate (Boletzky, 1989; Baeza and Fernández, 2002). Similarly, in marine vertebrates such as catfish, perch, and other fish, parents may display brood-care behavior, and this has an impact on the birth rate and bodily functions of fish larvae (Bunn et al., 2000; Baldridge and Lodge, 2013; Foster et al., 2016). The brood-care behavior affects the growth of *O. ocellatus* larvae and determines the immune and metabolic functions of the larvae to a certain extent (Zheng et al., 2009; Wang et al., 2010).

Metabolism is the synthesis and biochemical processes of cells and tissues (Harper and Patti, 2020). The metabolic network of organisms is very complex, and multiple metabolic processes share the metabolic functions together. Changes in one metabolic process often lead to changes in other processes. A good understanding of metabolic functions is conducive to the study of the physiological and biochemical functions of organisms (Hosseini et al., 2016; Matsumoto et al., 2018; Xu and Zheng, 2020). In studies of invertebrate metabolism, energy accounts for a large proportion; for example, glucose metabolism in marine mollusks or the comparison of energy metabolism between Bivalvia and Brachiopoda (Martínez-Quintana and Yepiz-Plascencia, 2012; Bruning et al., 2013; Payne et al., 2014). Studies of cephalopod metabolic impact factors mainly focus on external environmental factors, such as water depth and habitat temperature, while other studies consider metabolic mechanisms through starvation, individual size, and other aspects (Seibel et al., 1997; Pimentel et al., 2012; Ikeda, 2016; Speers-Roesch et al., 2016). The relationships between metabolism and brood-care behavior have not been reported up to now.

We divide *O. ocellatus* eggs laid by the same parent into Pro group (brood-care group) and Unp (brood-careless group) in our research. We performed transcriptome sequencing and bioinformatics analyses using these larvae in two groups at 0 h, 4 h, 12 h, and 24 h after hatching, including gene function annotation, differentially expressed genes analysis, GO enrichment analysis, KEGG functional enrichment analysis, and metabolism-related protein–protein interaction networks (PPI network) analysis. Among these analyses, 3,486 DEGs were identified, which may be related to metabolic processes. The results of a heatmap indicate that brood-care has a great impact on the metabolism of larvae. Ten metabolism-related KEGG signaling pathways were enriched in our study. They are closely related to larval metabolism and maintain the metabolic functions. We then construct a protein–protein interaction network using 49 genes enriched in these pathways and find multiple key genes most related to larval metabolism. In the end, quantitative RT-PCR (qRT-PCR) was used to identify and verify 10 hub genes. These results supply us with novel insights for further comprehending the relationships between *O. ocellatus* brood-care behavior and larval metabolic mechanisms. This conclusion lays a foundation for the artificial breeding technology of *O. ocellatus.*

## Materials and Methods

### Ethics Statement

*Octopus ocellatus* samples were obtained from a commercial hatchery. This research was conducted in accordance with the protocols of the Institutional Animal Care and Use Committee of the Ludong University (protocol number LDU-IRB20210308NXY) and the China Government Principles for the Utilization and Care of Invertebrate Animals Used in Testing, Research, and Training (State Science and Technology Commission of the People’s Republic of China for No. 2, October 31, 1988^1^).

### Sample Collection and RNA Preparation

We caught wild *O. ocellatus* parents in Rizhao sea area to prepare for this experiment, the latitude and longitude of animals sampling site are 34.91005177008973 and 119.63820016113283. The eggs spawned by parents were collected after temporary feeding, and these were divided into two groups (Pro and Unp). Among them, the eggs in Pro group continued to be brooded by their parents, and the eggs in Unp group were hatched in flowing seawater without parent brood. Then, the primary incubation larvae were temporarily cultured in floating seawater for 24 h. The temperature of seawater was stable at the optimum temperature of eggs hatching (19–20.8°C) in 29 days of hatching. The larvae at 0h, 4h, 12h, and 24h after hatching were collected and stored in liquid nitrogen container until RNA extraction using TRIzol method.

We randomly selected nine primary incubation larvae at each time point in two groups for RNA extraction: brood-care larvae grow for 0h (Pro-C), brood-care larvae grow for 4h (Pro-4h), brood-care larvae grow for 12h (Pro-12h), and brood-care larvae grow for 24h (Pro-24h); brood-careless larvae grow for 0h (Unp-C), brood-careless larvae grow for 4h (Unp-4h), brood-careless larvae grow for 12h (Unp-12h), and brood-careless larvae grow for 24h (Unp-24h). Three of the nine larvae in a group at each time point were randomly selected, and their equal molar mass of RNA was concentrated in a replicate as a template for constructing the transcriptome library; the same method was used to pooled equal molar mass RNA from the three larvae into the second replicate, and the equal molar mass RNA of the remaining larvae was concentrated in the third replicate. We subsequent qRT-PCR verification using remaining RNA that has been preserved.

### Library Construction and Illumina Sequencing

The method of Li et al. (2017) description was used to construct the library. And Illumina Hiseq 4000 platform was used to sequence samples.

### The Expression and Function Annotation of Genes

We splice Trinity with RSEM to obtain reference sequences and map the clean reads to these sequences. Then, a great quantity of read counts of each sample mapped to each gene was obtained. To understand the expression and abundance of genes, we transform read counts into FPKM. The result showed that there was a positive correlation between FPKM and the expression level of samples. The unigenes were annotated by searching the sequences against the NR, NT, GO, SwissProt, and KOG databases using BLASTX with a cut-off of Evalue ≤ 1e-5. Meanwhile, we annotated unigenes into Pfam database with Hmmer 3.0 package (Evalue ≤ 0.01).

### The Screening and Analysis of Differentially Expressed Genes

Differentially expressed genes (DEGs) were screened out using the DESeq2 package for R, and the filter parameters were | log2 Fold Change| ≥ 4 and q-value ≤ 0.01. We identified DEGs distribution and multiple GO terms by analyzing DEGs with GO functional enrichment analysis and conduct the DEGs statistical analyses. We used KEGG enrichment analysis to further study the specific functions of DEGs, and the metabolism-related pathways involved in DEGs were annotated. Finally, the signaling pathways with significant enrichment of DEGs were selected, and we count the species and number of DEGs after selecting.

### The Construction of Functional Protein Association Networks

STRING v11.0 with default parameters was used to construct PPI networks to study relationships between functions of metabolism-related genes (Damian et al., 2018).

### The Validation of Quantitative Real-Time Polymerase Chain Reaction

We verified the accuracy of our RNA-Seq results using quantitative real-time polymerase chain reaction (qRT-PCR) to validate 10 selected genes. In this experiment, each group contained three biological replicates, and Primer Premier 5.0 software was used to project gene-specific primers. We screened 10 genes for verification, and their names and primer sequences are listed in Table 1. The stability of three genes including *18S*, β*-actin*, and *GAPDH* were evaluated in different tissues and embryo development stages of *O. ocellatus*. By comparison, we used *O. ocellatus*β*-actin* as an endogenous control based on its expression level which tended to be stable. This experiment is based on Li et al.’s (2019) description method for qRT-PCR.

**TABLE 1:** Primer list for quantitative RT-PCR verification.

Gene name	Forward primer (5′-3′)	TM (°C)	Reverse primer (5′-3′)	TM (°C)	Amplicon length (bp)
*ADCY5*	GCAGTTTGAT GTGTGGTCTA	60	CCCAAATAGT TCAGGGTGTC	60	105
*ADCY9*	CAGTCCTCCT CTTCCCTATT	60	CACCTGAATG TCGTGTGTC	60	116
*AKT3*	CTCCACCGCA GACAATAAC	60	CTGCAGATGC GCTAAGATAG	60	129
*GRIA2*	CAGACAGACA GACACAGAGA	61	AGTAGTACTCC CTTGCTCATC	61	103
*GRM5*	CATCGTCTCA GATGGTCAAG	60	AGAGTCATGG GAGTCGTATC	60	112
*ITPR1*	GGCACCTTC CTTGTCTAAAT	60	AACGAGGCGA GAGAGTATTA	60	136
*MAP2K1*	CCATCAGAA AGGGCAGATTT	60	CTCCGGATCC ATACCCATTA	60	117
*PDE1A*	CCTTGGGCTG CTATTTCTATC	60	TCTGGGAGC CCATGTTTA	60	140
*PPP1R12A*	GTCGTTCAAC ACAGGGTATC	60	GTCAGAGCCCT TATCACATTC	60	119
*SLC8A2*	CACTCATCTT TCTCAGCAGTC	60	CTCGTGCTGGT GTTGATTAT	60	118

## Results

### Apparent Results of *Octopus ocellatus* Larvae

Brood-care behavior affects the hatching rate and survival rate of *O. ocellatus* larvae. The survival rate of brood-careless eggs was significantly lower than that of normal hatching larvae, and the hatching rate was reduced by 30–40%. However, there were no significant differences in morphology and development of larvae between Pro and Unp.

### The Results and Quality Assessment of Sequencing

Samples at four time points in two groups were sequenced by RNA-Seq. Table 2 shows the detailed results of sequencing. Raw sequencing reads were submitted to Sequence Read Archive in NCBI; the SRA accession numbers were SRR15204591, SRR15204592, SRR15204593, SRR15204594, SRR15204595, SRR15204596, SRR15204597, SRR15204598, SRR15204599, SRR15204600, SRR15204601, SRR15204602, SRR15927331, SRR15927332, SRR15927333, SRR15927334, SRR15927336, SRR15927337, SRR15927338, SRR15927339, SRR15927340, SRR15927341, SRR15927342, and SRR15927343^2^.

**TABLE 2:** Summary of sequencing results.

Sample	Raw reads	Clean reads	Q20 (%)	Q30 (%)
Pro-C-1	50,581,378	49,165,388	97.54	93.27
Pro-C-2	49,281,546	46,846,720	97.42	93.07
Pro-C-3	55,727,122	53,007,342	97.39	92.94
Pro-4h-1	43,683,716	40,502,546	97.38	92.93
Pro-4h-2	50,981,374	47,814,924	97.47	93.15
Pro-4h-3	44,781,414	41,833,784	97.30	92.78
Pro–12h-1	42,605,216	40,522,842	97.72	93.70
Pro-12h-2	54,564,714	51,170,578	97.30	92.72
Pro-12h-3	56,997,500	53,933,392	97.61	93.42
Pro-24h-1	51,418,618	49,235,968	97.71	93.67
Pro-24h-2	60,787,322	58,211,806	97.52	93.20
Pro-24h-3	63,795,232	61,219,086	97.57	93.32
Unp-C-1	69,297,090	66,526,390	97.51	93.22
Unp-C-2	49,510,168	47,736,620	97.41	93.01
Unp-C-3	52,262,354	49,721,676	97.36	98.89
Unp-4h-1	54,619,850	52,087,242	97.14	92.52
Unp-4h-2	62,608,192	59,723,836	97.26	92.76
Unp-4h-3	61,292,170	58,349,184	96.28	92.11
Unp-12h-1	64,476,180	61,827,626	97.47	93.17
Unp-12h-2	58,363,420	55,956,658	96.85	91.92
Unp-12h-3	65,401,084	62,469,082	97.42	93.08
Unp-24h-1	53,869,700	51,669,944	97.35	92.94
Unp-24h-2	49,403,688	45,089,092	97.22	92.70
Unp-24h-3	56,415,060	54,214,330	97.40	93.04

### Differential Expression Analysis

**FIGURE 1:**
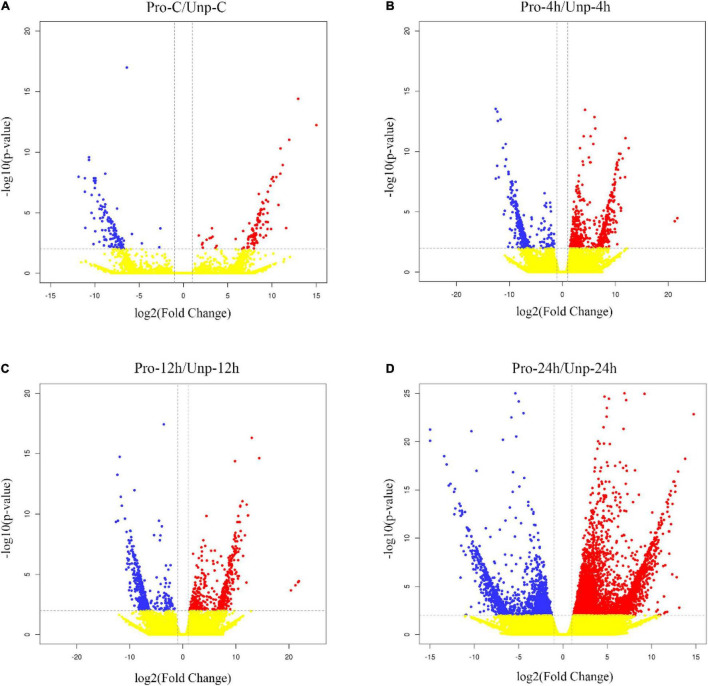
**(A)** Volcano plot of DEGs distribution trends between Pro-C and Unp-C. Each dot stands for a gene. Red dots indicate up-regulated DEGs; blue dots are down-regulated DEGs; and yellow dots stand for the genes with no difference. **(B)** Volcano Plot of DEGs distribution trends between Pro-4h and Unp-4h. **(C)** Volcano Plot of DEGs distribution trends between Pro-12h and Unp-12h. **(D)** Volcano Plot of DEGs distribution trends between Pro-24h and Unp-24h.

**FIGURE 2:**
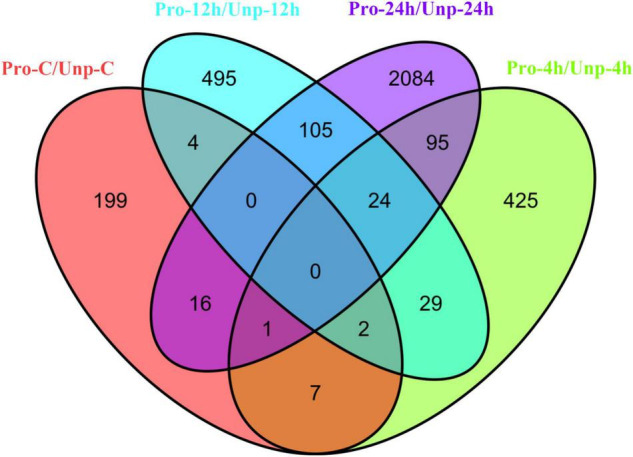
The Venn diagram shows the overlap of the DEGs at 0h (red), 4h (green), 12h (blue), and 24h (purple) after hatching.

**FIGURE 3:**
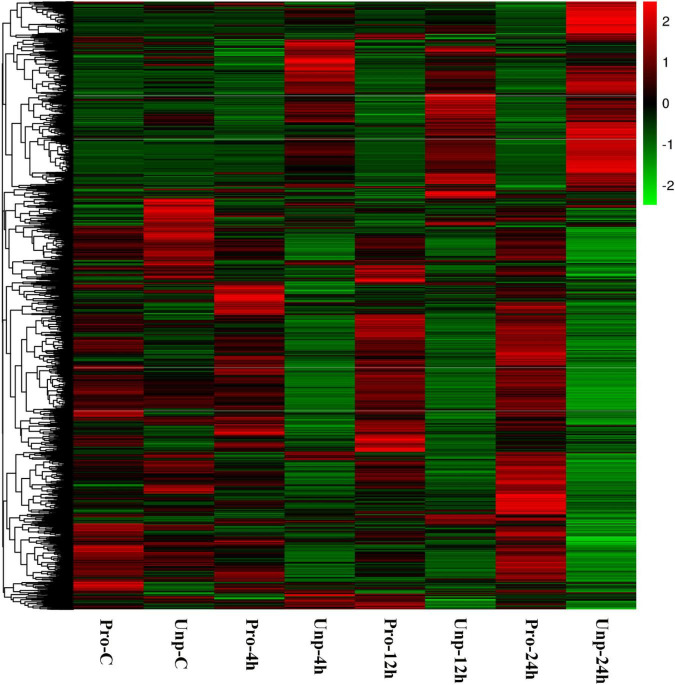
Heatmap analysis of hierarchical clustering of DEGs at four time points in two groups. Each row represents one gene, and each column stands for a time point. The colors range from green to red, indicating the level of expression from low to high.

Through differential expression analysis, we found 229, 583, 659, and 2,328 DEGs in Pro group at 0h, 4h, 12h, and 24h after hatching compared with Unp group. Among these, 101 DEGs were up-regulated, and 128 DEGs were down-regulated at 0h after hatching; 298 DEGs were up-regulated, and 285 DEGs were down-regulated at 4h after hatching; 323 DEGs were up-regulated, and 336 DEGs were down-regulated at 12 h after hatching; 1,657 DEGs were up-regulated, and 671 DEGs were down-regulated at 12 h after hatching (Figure 1). Venn diagram helps us find the union of DEGs at three time points (Figure 2). The clustering distribution of DEGs is shown in Figure 3 intuitively.

### GO and KEGG Enrichment Analyses of Differentially Expressed Genes

**FIGURE 4:**
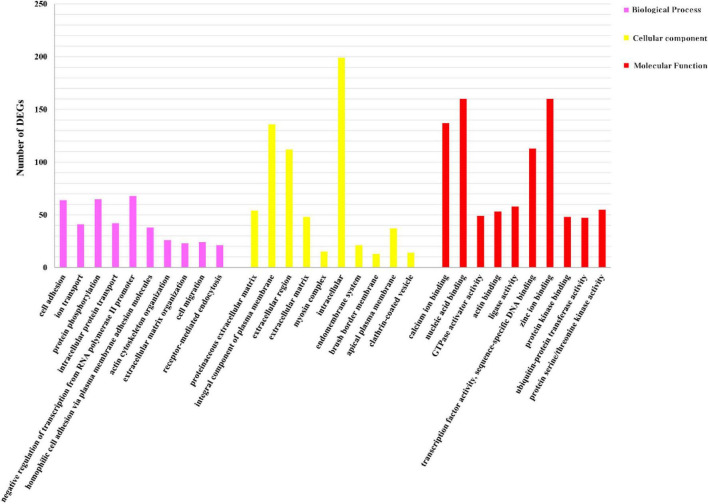
GO analysis of DEGs. Distribution of level-3 GO annotation in three categories. The *y*-axis represents the corresponding number of DEGs; the *x*-axis stands for the gene functional classification based on GO.

**FIGURE 5:**
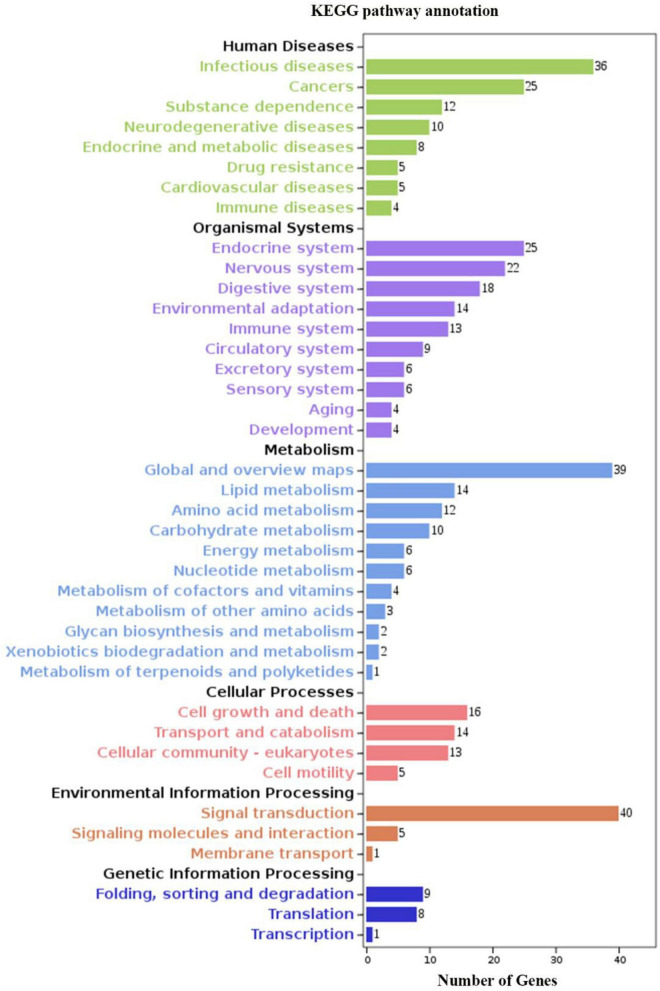
KEGG analysis of DEGs. The *y*-axis indicates level-2 KEGG classes; the *x*-axis represents the corresponding number of DEGs.

We identified 269 level-3 terms based on GO enrichment analysis including 151 biological process subclasses, 72 molecular function subclasses, and 46 cellular component subclasses. And the top 10 level-3 terms were shown in Figure 4. Meanwhile, The KEGG signaling pathways enriched with DEGs was analyzed to help us further understand the gene functions. We enriched 179 level-2 KEGG classes pathways containing 1,777 selected DEGs (Figure 5), and 10 significantly enriched metabolism-related pathways (Table 3) were identified in our study.

**TABLE 3:** Summary of 10 significant metabolism-related signaling pathways.

Pathways	Number of DEGs	p-value
Purine metabolism	8	0.002236
Long-term depression	5	0.004845
Glutamatergic synapse	6	0.016325
Vascular smooth muscle contraction	6	0.021284
Regulation of actin cytoskeleton	9	0.022174
cAMP signaling pathway	7	0.026442
Protein digestion and absorption	9	0.034676
Synaptic vesicle cycle	4	0.037186
Estrogen signaling pathway	6	0.041634
cGMP-PKG signaling pathway	7	0.045143

### Construction of Metabolism-Related Protein–Protein Interaction Networks

**FIGURE 6:**
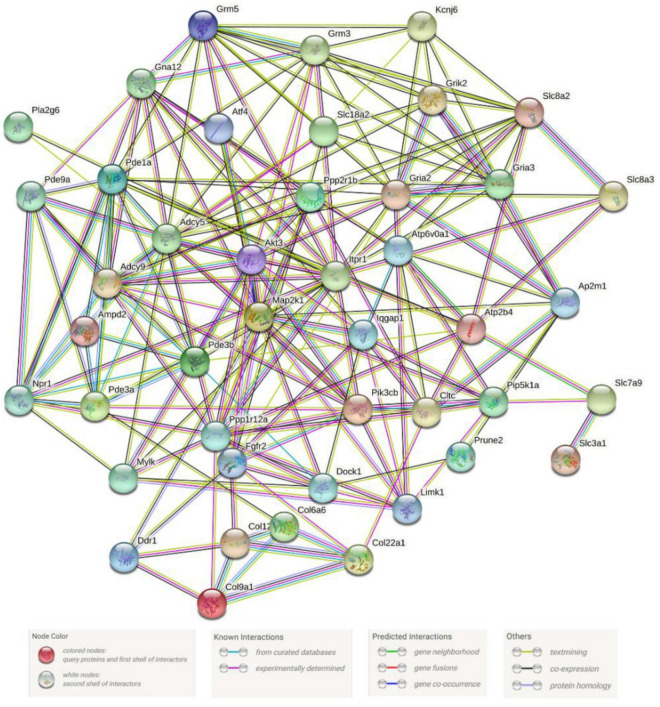
Metabolism-related protein–protein interaction networks. Network nodes stand for proteins. The legend represents the relationships between nodes.

Proteins exist in all organisms as the main components of cells and tissues. All significant parts of the organism need protein participation, which is the main undertaker of life activities. We can identify key genes in the metabolic gene population by constructing protein–protein interaction networks. In our research, we used 49 gene protein sequences in the signaling pathways in Table 3 to construct the protein–protein interaction networks. The networks were shown in Figure 6. We found that the ITPR1 gene had the highest number of protein-protein interactions, and other genes were interacted with more than 10 proteins. Relevant parameter information of them was listed in Table 4. There were 44 nodes and 197 edges in the network, and each node was interacted with an average of 8.95 nodes. The clustering coefficient was 0.5, and the *p*-value of this network was ≤ 1.0e-16.

**TABLE 4:** Network Statistics of metabolism-related proteins.

Network statistics	
Number of nodes	44
Number of edges	197
Average node degree	8.95
Clustering coefficient	0.5
Expected number of edges	86
PPI enrichment p-value	1.0E-16

### Analysis of Key Metabolism-Related Differentially Expressed Genes

The interaction relationships between key metabolism-related DEGs were studied in our research. We identified 10 genes (Table 5) with high protein interactions or involved in multiple signaling pathways based on KEGG enrichment and PPI networks analyses, and then explored their interaction relationships. We divided these 10 key DEGs into five categories: solute carrier family, purine metabolism signaling pathway, cAMP signaling pathway, protein digestion and absorption signaling pathway, and other significant metabolism-related DEGs. The family and signaling pathways were gone hand in hand to metabolic processes, analyzing them will promote us to further understand the metabolic mechanisms of differences of larval growth between Pro group and Unp group.

**TABLE 5:** Summary of 10 key DEGs.

Gene name (abbreviation)	Gene name (official full name)	Number of protein–protein interactions	Number of KEGG signaling pathways
*ADCY5*	adenylate cyclase type 5	19	13
*ADCY9*	adenylate cyclase 9	15	8
*AKT3*	AKT serine/threonine kinase 3	14	12
*GRIA2*	glutamate receptor, ionotropic kainate 2	15	6
*GRM5*	glutamate metabotropic receptor 5	12	6
*ITPR1*	inositol 1,4,5-trisphosphate receptor 1	21	8
*MA2PK1*	mitogen activated protein kinase kinase 1	19	7
*PDE1A*	phosphodiesterase 1A	13	3
*PPP1R12A*	protein phosphatase 1, regulatory subunit 12A	14	5
*SLC8A2*	solute carrier family 8 member A2	12	2

### Differentially Expressed Genes Verification by Quantitative Real-Time Polymerase Chain Reaction

**FIGURE 7:**
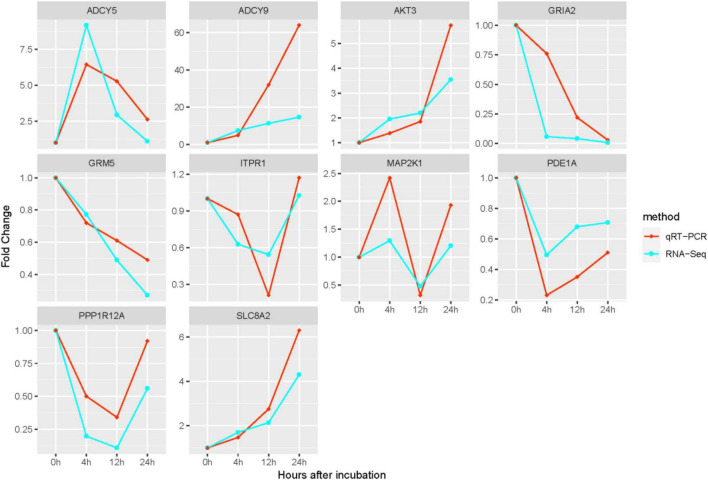
Comparison of expression of 10 hub genes between qRT-PCR and RNA-Seq results. The transcript expression levels of the selected DEGs were each normalized to that of the β*-actin* gene. The *x*-axis represents the growth time after hatching; the *y*-axis stands for fold change.

We detected the expression of 10 metabolism-related DEGs at each time point in the two groups using qRT-PCR, and verified the consistency of qRT-PCR and RNA-Seq expression. The results of qRT-PCR suggested that all DEGs measured were single products. We compared the gene expression profiles between qRT-PCR results and RNA-Seq results (Figure 7). And the result suggested that their results were significantly correlated, and showed the same trend pattern.

## Discussion

### The Purpose and Significance of This Study

Due to the rich nutrition and excellent taste, *O. ocellatus* is gradually favored by people (Boletzky, 1975; Wei et al., 2018). As an economically important species employed in mariculture, the artificial breeding of *O. ocellatus* is an issue of concern. We understand that multiple octopuses have the brood-care behavior based on previous studies, and the behavior directly affects the growth and development of their larvae. Thus, understanding the effects of brood-care behavior is vital to *O. ocellatus* larval growth and artificial breeding. Our research screened 3,486 DEGs, and these genes are considered to have significant relationships with *O. ocellatus* larval metabolic processes. A heatmap showed that the genes in two groups have significant differences of the same point in time. It is further indicated that there are quite metabolic differences between the brood-care larvae and the brood-careless larvae. Then, we identified 10 metabolism-related KEGG signaling pathways, they interact with each other and share the metabolic function of organisms. Finally, we construct protein-protein interaction networks using 49 DEGs in these signaling pathways to further explore *O. ocellatus* larval metabolic mechanisms.

### Enrichment Analyses of Metabolism-Related GO Terms and KEGG Pathways

Many metabolism-related terms and pathways were yielded through GO and KEGG enrichment analyses, including cell adhesion term, protein phosphorylation term, cAMP signaling pathway, purine metabolism signaling pathway, and other key pathways and terms. These pathways and terms are directly related to metabolic processes, suggesting that there were several metabolism-related DEGs between larvae in two groups, and thus resulted in complex metabolic mechanisms of larvae. We comprehensively analyzed these terms and signaling pathways. The results will help us to understand the metabolic processes and molecular mechanisms of *O. ocellatus* under different incubation conditions within 24h of incubation, so as to lay a foundation for the artificial breeding of *O. ocellatus*.

### Speculation of Hub Genes

Protein is an essential component of an organism and is closely related to metabolic processes. Studying their interaction relationships helps us to enrich our understanding of biological metabolic mechanisms We construct protein–protein interaction networks based on 49 key genes contained in metabolism-related signaling pathways. These proteins interacted significantly better than randomly selected groups of proteins of similar size. This conclusion suggests that the above proteins may function as a group. We thus speculated that proteins interact with more other proteins in the network were hub proteins in metabolism. The genes corresponding to hub proteins are further studied.

### Functional Analyses of Metabolism-Related Hub Genes and Signaling Pathways

In our research, we analyze the differences in metabolic mechanisms between larvae under different hatching conditions using transcriptome profiling. The results help us to further understand the effect of *O. ocellatus* brood-care behavior on metabolic mechanisms. Ten hub genes were speculated and screened involved in multiple protein interactions or metabolism-related signaling pathways, and these hub genes, pathways, and the larval metabolic differences between two groups at the same time point were investigated.

### Analysis of Three Metabolic Pathways

Purine metabolism is one of the key ways for aquatic animals to detoxify and obtain energy, which plays a major part in maintaining the metabolic stability of organisms. In the liver of marine organisms, purines are converted by degrading enzymes into urea and glyoxylate, and then excreted by other metabolic pathways (Noguchi et al., 1982). In recent studies, we found that there is a urease that can decompose urea into NH3 in marine invertebrates. NH3 is very soluble in water, organisms can excrete urea in the form of NH3 and CO2 through purine metabolism to prevent poisoning caused by excessive urea in the body (Yeldandi et al., 1996). In addition to detoxification, purine metabolism will release ATP, ADP, and other derivatives, providing the energy necessary for biological life activities (Furchgott, 1966; Sakuraba et al., 1996). In our research, key metabolism-related genes such as NPR1 and AMPD2 were enriched in this pathway. Compared with the Unp group, NPR1 was continuously up-regulated with the growth of *O. ocellatus* larvae, AMPD2 was down-regulated slightly in the first 4 h of growth, and up-regulated significantly in the last 20 h. These results indicate that brood-care larvae have higher detox and energy production abilities than brood-careless larvae, and that they have higher metabolic capacity; cAMP is an essential substance in biological cells. It regulates many biological functions, such as proliferation, differentiation, and apoptosis of cells. Meanwhile, cAMP also plays a role in regulating the balance of energy and nutrition. cAMP is widely distributed in many organelles, such as nucleus and cytoplasm. In mitochondria, cAMP can stabilize the function of mitochondria, and promote the growth of *O. ocellatus* larvae by regulating the stress response of cells and mitochondrial activity (Zhang et al., 2016). cAMP signaling pathway acts a key part in regulating the metabolism of saccharides and fat. It maintains the metabolic homeostasis by regulating the content of various saccharides. At the same time, it can regulate the formation of fat and the distribution of lipid in adipose tissue, control the differentiation of adipocytes, and maintain the fat content *in vivo* (Ravnskjaer et al., 2015). The maladjustment of cAMP signaling pathway may lead to cell metabolic disorders and the occurrence of various diseases (Tang et al., 2021). In our research, a large number of DEGs were significantly enriched in this pathway. And most DEGs were up-regulated in Pro group compared with Unp group, suggesting that the metabolic mechanisms of the brood-care larvae were more complex, and the ability to regulate this metabolic process was stronger. This phenomenon explains that the *O. ocellatus* larvae in Pro group had a stronger metabolic capacity and higher survival rate compared with those in Unp group; Protein digestion and absorption signaling pathway regulates the decomposition of proteins by digestive organs to produce favorable carbohydrates and energy (Freeman and Kim, 1978). This pathway was significantly enriched in our research, indicating that the digestive organs of *O. ocellatus* larvae were active, and metabolized rapidly. Moreover, we found multiple metabolism-related genes enriched in this pathway were significantly up-regulated compared with brood-careless larvae, including COL22A1, COL12A1, and other key genes. However, COL9A1 were down-regulated in our research, the specific reasons need to additional explore. All of these pathways are important pathways for the regulation of biological metabolism, and they are significantly enriched. Compared with the Unp group, most of the genes that are beneficial to biological metabolism enriched in above three important pathways were up-regulated, which suggests that the metabolic efficiency of *O. ocellatus* larvae in the Pro group was higher and more stable. And it was beneficial to the growth and development of *O. ocellatus* larvae.

#### Solute Carrier Family

Solute carrier families are the main factors affecting solute transport in organisms. There are many subunits in the family, and they act roles in almost all organs. In this study, we significantly enriched five family members, which play a significant regulatory part in substance transport and metabolism. SLC8A2, also known as NCX2, is a plasma membrane transporter that regulates intracellular calcium concentration and plays a major part in the regulation of Ca2+ in stomach, intestine, vascular smooth muscle, and other organs (Azuma et al., 2012). Similarly, as another subtype of the solid carrier family, SLC8A3 is highly expressed in the brain, and can regulate the concentration of Ca2+ in the excitatory cells. It is worth mentioning that SLC8A3 also acts a key role in maintaining blood transport in the brain (Sokolow et al., 2004; Yuan Z. et al., 2016). Ca2+ is an essential part of biological metabolism processes, which can regulate the synthesis and release of neurotransmitters and hormones. Above two genes regulate the concentration of Ca2+, indicating that they are important genes regulating the metabolic process (Wu et al., 2020). SLC3A1 and SLC7A9 are cystine transport factors, they regulate the metabolic processes of organisms by regulating the concentration of cysteine and the synthesis of glutathione (Alter et al., 2016). As an ammonia transport factor, SLC18A2 is active in a variety of organisms. Lack of SLC18A2 will result in obstruction of the motor system, which will affect the larvae from normal activities (Jin et al., 2014). The five genes of solute carrier family enriched in our study were all metabolism-related, and their high expression was helpful to *O. ocellatus* larval growth and development. Beside SLC3A1, the other four genes were significantly up-regulated in brood-care larvae compared with brood-careless larvae, indicating that larvae in the Pro group had more complex metabolic processes and higher metabolic capacity.

#### Three Significant Hub Genes Analysis

Among all hub genes, ITPR1, MAP2K1, and ADCY5 were enriched in more than seven metabolism-related signaling pathways, and they possessed higher numbers of protein–protein interactions. ITPR1, also known as IP3R1, is a phosphate receptor that regulates the concentration of Ca2 +, and promotes the metabolic activity of organisms by increasing the concentration of Ca2+ (Wakai and Fissore, 2019). In somatic cells, Ca2 + is transported to mitochondria to stimulate mitochondrial metabolism. And in oocytes, Ca2 + can increases cell activity and ATP content (Valladares et al., 2018; Wakai and Fissore, 2019). Moreover, ITPR1 plays a significant part in regulating vasodilation and maintaining normal blood pressure (Yuan Q. et al., 2016). MAP2K1 is a protein kinase, which exists in most cells, and can be activated by many extracellular signals to regulate metabolism processes of organisms (Crews et al., 1992). MAP2K1 can regulate cell growth, proliferation, and differentiation, and maintain the stability of biological metabolism (Chung et al., 2011). ADCY5 is an important subtype of adenyl cycles family, which transforms ATP into cAMP *in vivo*. As a protein kinase activator, cAMP is widely used in various organisms. It regulates the physiological function and material metabolism to control cell activity and hormone application, which plays an important part in carbohydrate, fat metabolism, nucleic acid, and protein synthesis (Richter-Landsberg and Jastorff, 1986; Frezza et al., 2018). The above three genes are significantly enriched, and have the highest protein interaction number, which are closely related to metabolism. Compared with the Unp group, the expression of ADCY5 gene was up-regulated at 4h after hatching, and down-regulated from 4 to 24 h in the Pro group; the expression of ITPR1 was down-regulated in the first 12 h, and significantly up-regulated in the second 12 h; the expression of MAP2K1 was down-regulated from 4h to 12h, and significantly up-regulated within 0 to 4 h and 12 to 24 h, indicating that the brood-care eggs had more complex metabolic mechanisms. These results explain that the *O. ocellatus* larvae in Pro group had a stronger metabolic capacity compared with those in Unp group. However, the specific functions of these genes in larval metabolism need to be further explored.

#### Other Signaling Pathways and Hub Genes

In addition to the above signaling pathways and hub genes, other metabolism-related key genes and signaling pathways involved in the metabolism of *O. ocellatus* larvae were identified such as long-term depression signaling pathway, glutamatergic synapse signaling pathway, and two key genes AKT3 and ADCY9. In previous studies, these two genes and signaling pathways have been reported to be involved in biological metabolic processes and act significant roles (Marty and Llano, 1995; Rodríguez and Ortega, 2017; Teixeira et al., 2017; Chen et al., 2018; DuBois et al., 2019). The effects of genes and pathways on metabolism of *O. ocellatus* under different brood-care behaviors need to be further explored from now on.

## Conclusion

We performed transcriptome profiling of gene expression in larval growth from brood-care and brood-careless eggs and constructed a PPI network. Ten hub genes with numerous protein–protein interaction relationships or that involved in multiple KEGG signaling pathways were identified. In our research, we first study the effects of brood-care behavior on metabolic mechanisms of *O. ocellatus* larval growth using PPI networks. Our results provide valuable gene resources for understanding the metabolism of invertebrate larvae. Meanwhile, the data lays a foundation for further understanding invertebrate brood-care behaviors.

## Data Availability Statement

The original contributions presented in the study are publicly available. These data can be found here: SRR15204591–SRR15204602, SRR15927331–SRR15927334, and SRR15927336–SRR15927343.

## Ethics Statement

The animal study was reviewed and approved by the Institutional Animal Care and Use Committee of the Ludong University (protocol number LDU-IRB20210308NXY) and the China Government Principles for the Utilization and Care of Invertebrate Animals Used in Testing, Research, and Training (State Science and Technology Commission of the People’s Republic of China for No. 2, October 31, 1988; http://www.gov.cn/gongbao/content/2011/content_1860757.htm).

## Author Contributions

ZL and JY designed and supervised the study. XB, XL, BY, YL, MC, and BL prepared the samples. XB, XL, YF, GS, WW, and XX analyzed all sequencing data. XB and ZL wrote the manuscript. All authors have read and approved the final manuscript.

## Conflict of Interest

BL was employed by the company Yantai Haiyu Marine Science and Technology Co. Ltd. The remaining authors declare that the research was conducted in the absence of any commercial or financial relationships that could be constructed as a potential conflict of interest.

## Publisher’s Note

All claims expressed in this article are solely those of the authors and do not necessarily represent those of their affiliated organizations, or those of the publisher, the editors and the reviewers. Any product that may be evaluated in this article, or claim that may be made by its manufacturer, is not guaranteed or endorsed by the publisher.

## Footnotes

1
http://www.gov.cn/gongbao/content/2011/content_1860757.htm

2
https://www.ncbi.nlm.nih.gov/Traces/study/?acc=SRP329195&o=acc_s%3Ad
